# Prescription medication use during pregnancies that resulted in births and abortions (2001-2013): A retrospective population-based study in a Canadian population

**DOI:** 10.1371/journal.pone.0211319

**Published:** 2019-03-06

**Authors:** Christine Leong, Dan Chateau, Matthew Dahl, Jamie Falk, Alan Katz, Shawn Bugden, Colette Raymond

**Affiliations:** 1 College of Pharmacy, Rady Faculty of Health Sciences, University of Manitoba, Winnipeg, Manitoba, Canada; 2 Manitoba Centre for Health Policy, Department of Community Health Sciences, University of Manitoba, Winnipeg, Manitoba, Canada; the University of Sydney, AUSTRALIA

## Abstract

We aimed to describe medication use in pregnancies that resulted in births and abortions, as well as use after a pregnancy-related visit to characterize the receipt of medication after knowledge of pregnancy. Abortions included both spontaneous and induced abortions. Rates of medication use among women with a pregnancy outcome (2001–2013) were described using the Manitoba Population Research Data Repository at the Manitoba Centre for Health Policy. Use was determined as ≥ 1 prescription filled during pregnancies that resulted in births (livebirth/stillbirth) and abortions. Rates were calculated at any time during pregnancy and after a pregnancy-related visit. Rates were additionally characterized by risk in pregnancy using Briggs classification (2017). Of 174,848 birth pregnancies, overall 64.9% filled ≥ 1 prescription during pregnancy (a significant increase from 62.3% to 68.8% from 2001–2013, p<0.0001); 55.4% filled ≥ 1 prescription after a pregnancy-related visit. Of 71,967 abortions, 44.7% filled ≥ 1 prescription (a significant increase from 42.6% to 46.8% from 2001–2013, p<0.0001). Only 3.7% of birth pregnancies had at least one prescription for a contraindicated medication (according to Briggs classification), whereas 10.8% of abortions filled a prescription for a contraindicated medication. The most common drugs used in pregnancy were amoxicillin, doxylamine, codeine combinations, nitrofurantoin, cephalexin, salbutamol and ranitidine. Fewer women filled prescriptions for undesirable medications according to Briggs classification during pregnancy after a pregnancy-related visit.

## Introduction

Drug prescribing in pregnancy remains a complex and controversial issue for pregnant women and clinicians [1–3]. As the availability and use of medications change over time, understanding the real world use of prescription medications during pregnancy is imperative to assessing exposure and risk at a population level [3].

While several studies have evaluated prescription drug use in large populations [1–9], wide variation in drug use estimates during pregnancy exists [3] and limited data describes North American prescribing patterns [1,3–5]. Many of these studies only use gestational age estimates [1,6] and few population-based studies have assessed prescription drug use during pregnancy [4,5,7]. Studies that describe medication use after a pregnancy-related healthcare visit to approximate intentional use during pregnancy [6,8] or pattern of drug use in aborted pregnancies [9] are also limited.

The risk assessment of medications used in pregnancy has traditionally been guided by the United States (US) Food and Drug Administration (FDA) classification system, which up until June 30, 2015 classified the potential risk of drug exposure to the developing fetus into five categories (A, B, C, D, and X) based on the type of available data. These risk categories continue to evolve [10]; however, this system can be misleading because there is a lack of safety data in humans for the majority of medications. As a result, many medications are categorized as FDA Category C where use in pregnancy is determined by weighing the potential benefit against the potential risk, which could impact clinical practice and research. It should be noted that after June 30, 2015, the U.S. FDA developed a new labeling system to provide general information on risk during pregnancy, clinical considerations, and level of evidence to support the risk statements [11,12]. However, this will only be applied to medications approved from 2001 onwards 11,12]. The Briggs’ Drugs in Pregnancy and Lactation (2017) [13] textbook of medication use in pregnancy is an alternative evidence-based classification system that is used in clinical practice and by several studies [6]. This reference has 17 pregnancy recommendation categories of risk based on the type of available data and trimester of exposure, which can help provide further clinical context into decision-making (**S2 Table**).

To date, there are no large Canadian database studies that assess the use of medications in pregnancy after a pregnancy-related visit to a health professional, or for pregnancies that resulted in abortion. Furthermore, no studies to date have described the use of medications in pregnancy using the Briggs’ risk criteria. We examined the use of medications before, during, and after pregnancy, as well as after a pregnancy-related health care visit in Manitoba, Canada. This study also described the population risk of drug exposure using the Briggs’ criteria for risk in pregnancy.

## Methods

### Data sources

This retrospective cohort study used the Manitoba Population Research Data Repository of administrative health data housed at the Manitoba Centre for Health Policy, which includes de-identified linked data for over 95% of the 1.2 million individuals living in Manitoba. These data include administrative information for the universal healthcare system for nearly all Manitobans, are linkable at the person-level using an encrypted identifier, and have been validated and used in health services research [14,15]. The Manitoba Health Insurance Registry provided demographic information for the population. Prescription drug data was obtained from the Drug Program Information Network (DPIN), which captures the drug name and quantity of medication dispensed from community pharmacies in Manitoba for all Manitobans regardless of drug insurance coverage. Receipt of medication is captured within DPIN regardless of whether the prescriber phones in the prescription or if the prescription is handed in person or faxed to the pharmacy. Physician claims data and hospital discharge abstract data were used to identify diagnoses and pregnancy outcomes by International Classification of Disease (ICD) diagnosis codes. Hospital records also provided information on the gravidity, parity, and gestational age for each birth. Socioeconomic status was determined by the neighborhood income quintile available through Statistics Canada census public use files. Dissemination Area level average household income values from the public-use Census files are used to construct the quintiles.

### Study population

All women with a pregnancy outcome identified through hospital discharge records and medical claims data [16] and continuous healthcare coverage from 365 days preconception to 90 days post-partum between the calendar years 2001 and 2013 were included. Pregnancy outcomes were identified through hospital discharge records and medical claims data included: (1) livebirth, stillbirth, or intrauterine death (ICD9CM V27, 656.4, ICD10 Z37, O36.4); and (2) spontaneous and induced abortion ICD9CM 632, 634–637, ICD10 O03-O05, ICD9-CM procedure codes 6901, 6951, 7491, 750, ICD-10 procedure code 5.CA 88–90). Women with molar or ectopic pregnancies were excluded. Due to uncertainty around delivery date, women with a hospital length of stay for more than seven days and no newborn data to assign a birthdate (and therefore, gestational age) were excluded [4]. A pregnancy-related visit to a health professional included prenatal care visits and diagnostic codes related to the pregnancy, labor, and/or delivery (with Manitoba tariff code 8400 or 8401 or diagnosis code of ICD-9-CM 640–648, 650–659, 660–669, V22, V23) [16]. Gestational age was available through hospital discharge data (maternal and newborn records) for 98.6% of livebirths/stillbirths, the remainder of which were excluded in the analysis. For abortions, gestational age from hospital discharge or medical claims data was used if available, if not, the earliest date of any pregnancy-related visit to a health care professional [16] (with Manitoba tariff code 8400 or 8401 or diagnosis code of ICD-9-CM 640–648, 650–659, 660–669, V22, V23) was used as the best estimate of the date of conception. If neither were available, the date of conception was assumed to be eight weeks prior to abortion procedure data, based on the definition used in previous research [9,17]. For abortions, pregnancy end date was based on the admission record for the first occurrence of an abortion procedure or medical claim. Readmissions with an abortion event within 28 days of the first abortion event were excluded [9]. Pregnancies resulting in multiples were included as a single pregnancy and women with multiple pregnancies were included as multiple pregnancy observations. Date of conception was determined by subtracting the gestational age (in weeks) from the date of maternal hospital admission [4]. First trimester was determined as date of conception plus 91 days, second trimester plus 92–182 days and third trimester was plus 183 days through date of pregnancy outcome.

### Medication exposure

Use of medication was defined as at least one filled prescription identified from the DPIN [4,5,9]. Medication use during pregnancy was calculated separately for pre-pregnancy (defined as use during the 365 days pre-conception), each trimester of pregnancy, the entire pregnancy period (defined as use between date of conception and pregnancy outcome), and three months after the pregnancy outcome. A medication dispensation after a pregnancy-related visit to a health professional [prenatal care visit (Manitoba tariff code 8400 or 8401) or diagnosis code of ICD-9-CM 640–648, 650–659, 660–669, V22, V23) defined intentional medication use in pregnancy. The World Health Organization Anatomical Therapeutic Chemical (ATC) drug classification system was used to categorize medications by drug class [18]. Medications were also classified according to Briggs’ Drugs in Pregnancy and Lactation (S2 Table) [13]. For prescriptions with combinations products, exposure was placed in the highest risk category. For oral solid doses that were combination drug products where the systemic medication was available for more than one separate products, this was assessed as multiple products. Exposure during pregnancy was based on the date that the prescription was filled at an outpatient pharmacy. Over-the-counter medications, even if provided by prescription, as well as vitamins and minerals were excluded. For medications classified as contraindicated or as associated with risk according to Briggs et al, the rates of pregnancies with exposure to these medications was characterized excluding female reproductive hormones (contraceptives, medications to stimulate ovulation, estrogens, progesterones and other fertility drugs) [6]. Medications were also classified as undesirable according to Briggs categories 10 to 17 (S2 Table), which indicate that human data exist that suggest risk in pregnancy or contraindicated in pregnancy.

### Statistical analysis

Descriptive statistics with 95% confidence intervals (CIs) on prevalence of use of medications during pregnancy is presented. All data management, programming, and analyses were performed using SAS statistical analysis software, version 9.4 (SAS Institute Inc., 2011). Chi-squared tests were used to test for time trends. Where applicable, poisson regression models were used to compare the percent of weighted pregnancies over the study period since not all women completed all trimesters. The weighted value was calculated by summing over all pregnancies, the actual number of days each woman contributed during a time period and that by the possible number of days in that time period (e.g. 91 days for each trimester).

## Ethics

This study was approved by the University of Manitoba Human Research Ethics Board.

## Results

There were a total of 174,848 (70.8%) birth pregnancies between 2001 and 2013, of which, 173,680 (99.3%) resulted in a livebirth and 1,168 (0.7%) resulted in a stillbirth. The mean gestational age for birth pregnancies was 38.93 weeks (SD 2.24 weeks). There were 71,969 (29.2%) abortions that were identified, in which a gestational age was available from health records (i.e., hospital, physician claims, and pregnancy-related visits) for 15,766 (21.9%). The gestational age was estimated to be eight weeks for the remaining pregnancies that resulted in abortion 56,201 (78.1%). The mean gestational age for abortions was 8.56 weeks (SD 2.21 weeks), which includes the eight-week imputed estimate. **Table 1** describes the characteristics of the study population. A higher proportion of women under 25 years and over 40 years of age had a pregnancy resulting in an abortion compared to women with a pregnancy resulting in birth (43.0% vs. 30.2% and 4.6% vs. 2.2%, respectively). A higher proportion of women with a pregnancy resulting in an abortion resided in the lowest income quintile neighborhood compared to the proportion of women with a pregnancy resulting in a livebirth (29.4% vs. 26.3%).

**Table 1:** Characteristics of the population.

	Overall (N = 246,817)	Birth (N = 174,848)	Abortion (N = 71,969)
Maternal Age at Delivery (N,%)			
< 25 Years	83824 (33.96)	52862 (30.23)	30962 (43.02)
25–29 Years	68678 (27.83)	51850 (29.65)	16828 (23.38)
30–39 Years	87124 (35.30)	66279 (37.91)	20845 (28.96)
40+ Years	7191 (2.91)	3857 (2.21)	3334 (4.63)
Maternal Age at Delivery (years) (Mean, SD)	27.52 (6.24)	27.84 (5.90)	26.76 (6.96)
Gestational Age (N,%)			
<20 Weeks	71632 (29.02)	51 (0.03)	71581 (99.46)*
20–36 Weeks	13859 (5.62)	13456 (7.70)	388 (0.54)*
37+ Weeks	161326 (65.36)	161341 (92.27)	0 (0.00)
Gestational Age (weeks) (Mean, SD)	30.08 (13.98)	38.93 (2.24)	8.56 (2.21)*
Number of Past Pregnancies (including current one) (N,%)			-
Missing	72169 (29.24)	201 (0.11)	71968 (100)
1 Pregnancy	51236 (20.76)	51236 (29.30)	N/A
2 Pregnancies	49925 (20.23)	49925 (28.55)	N/A
3 Pregnancies	31192 (12.64)	31192 (17.84)	N/A
4 Pregnancies	17755 (7.19)	17755 (10.15)	N/A
5 Pregnancies	10025 (4.06)	10025 (5.73)	N/A
6+ Pregnancies	14515 (5.90)	14515 (8.34)	N/A
Number of Past Pregnancies (including current one) (Mean, SD) (Missing Excluded)	2.71 (1.91)	2.71 (1.91)	N/A
Number of Past Deliveries (N,%)			-
Missing	72149 (29.23)	184 (0.11)	71965 (99.99)
0 Deliveries	65814 (26.67)	65811 (37.64)	s**
1 Delivery	55963 (22.67)	55963 (32.01)	N/A
2 Deliveries	27468 (11.13)	27468 (15.71)	N/A
3 Deliveries	12318 (4.99)	12318 (7.04)	N/A
4 Deliveries	6086 (2.47)	6085 (3.48)	N/A
5 Deliveries	3327 (1.35)	3327 (1.90)	N/A
6+ Deliveries	3692 (1.50)	3710 (2.13)	N/A
Number of Past Deliveries (Mean, SD) (Missing Excluded)	1.24 (1.76)	1.24 (1.76)	N/A
Days Difference between Conception Date and First Pregnancy-related Physician Visit (Median, IQR)	67 (48–90)	72 (52–93)	39 (24–51)
Income Quintile at Delivery (N,%)			
Income Quintile 1 (lowest)	67152 (27.21)	45968 (26.29)	21184 (29.43)
Income Quintile 2	51407 (20.83)	35802 (20.48)	15605 (21.68)
Income Quintile 3	45345 (18.37)	32377 (18.52)	12968 (18.02)
Income Quintile 4	44001 (17.83)	32237 (18.44)	11764 (16.35)
Income Quintile 5 (highest)	37947 (15.37)	27825 (15.91)	10122 (14.06)
Income Quintile Not Found	965 (0.39)	639 (0.37)	326 (0.45)

* Gestational age was available for 15,766 (21.9%) and missing in 56,201 (78.1%) of pregnancies resulting in abortion. Gestational age was assumed to be eight weeks for abortions where gestational age information was missing.

**s represents <6 people to comply with privacy policy

**S1 Table** lists the rates of prescriptions by the World Health Organization Anatomical Therapeutic Chemical Level 1 during pregnancy overall and after a pregnancy-related visit. The most frequently filled medication classes during pregnancy were antinfectives for systemic use (41.3% of all pregnancies) and respiratory medications (28.0% of all pregnancies).

**Fig 1:**
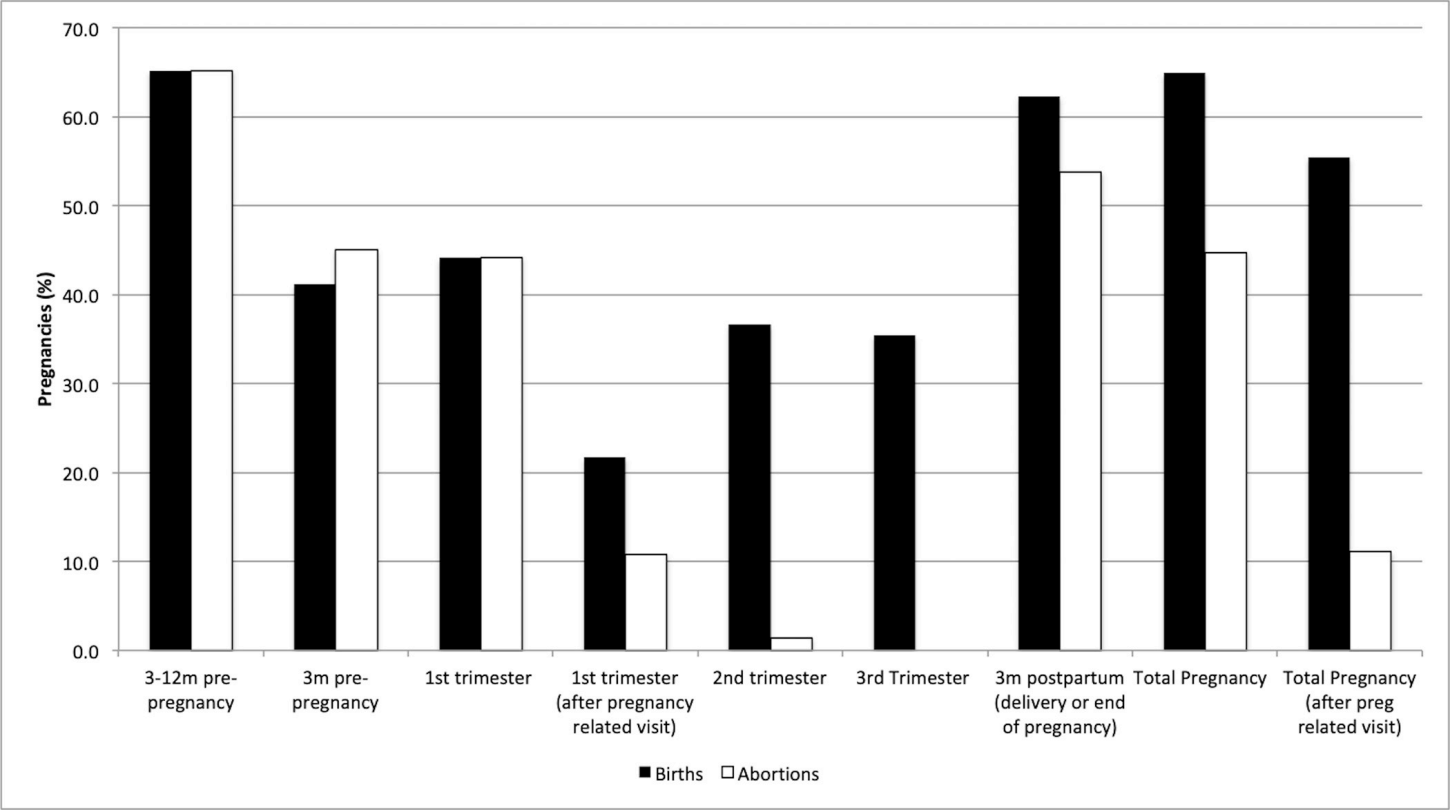
Pregnancies involving the receipt of at least one prescription medication, by time period and pregnancy outcome (2001–2013). *births, N = 174,848; abortions, N = 71,969.

**Fig 2:**
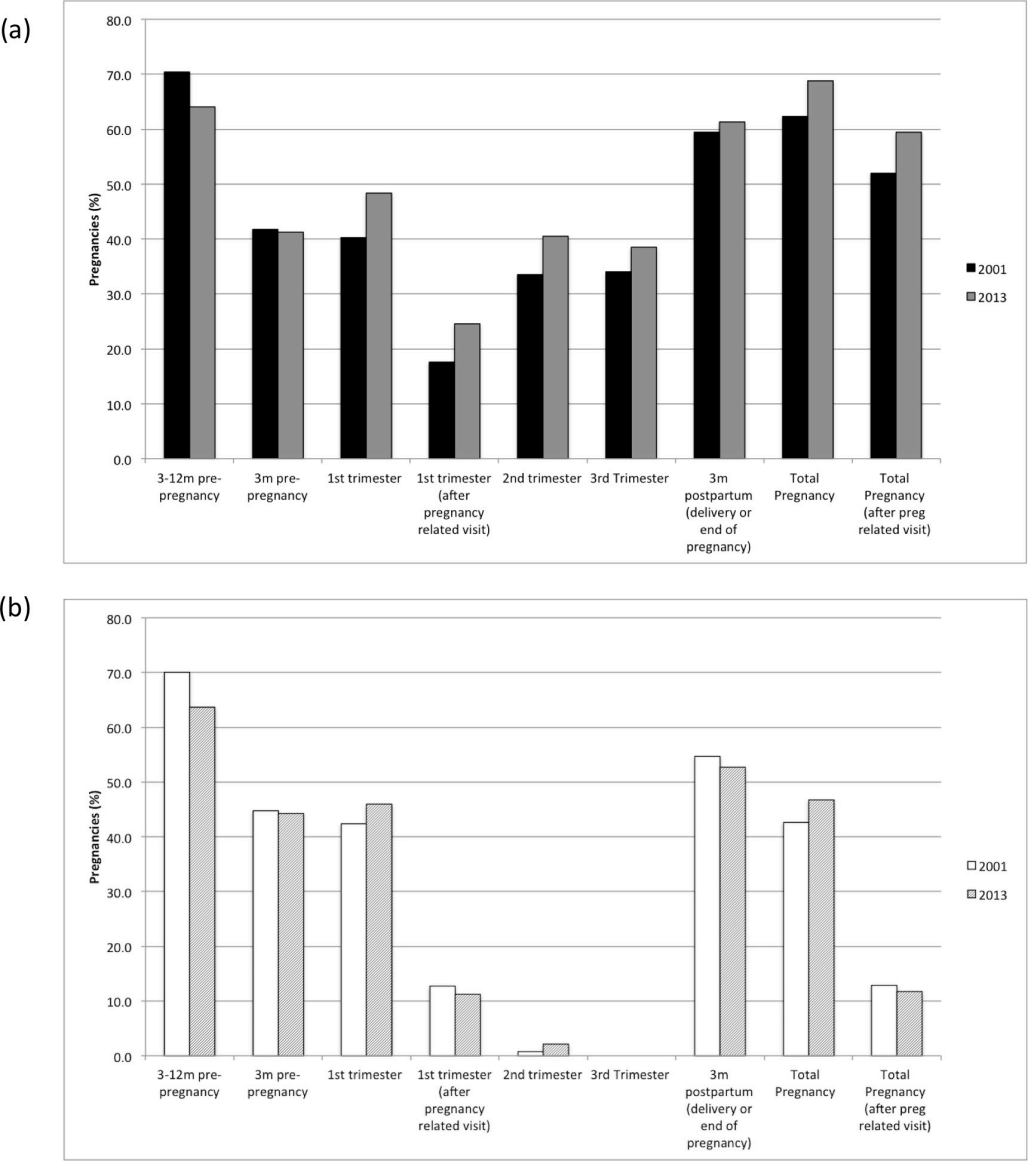
(a) Births (livebirth/stillbirth) and (b) Abortions involving the receipt of at least one prescription medication during pregnancy, by time period and year.

Overall, 64.9% of pregnancies that resulted in a birth received at least one prescription medication over the entire study period (**Fig 1**). This proportion dropped to 55.4% (p<0.0001) when the prescription was filled after a pregnancy-related visit to a health professional. In contrast, 47.7% of pregnancies that ended in abortion had a prescription filled. There was no difference between the proportion of pregnancies that resulted in abortions and births that received at least one prescription in the first trimester (44.1% and 44.2%, respectively, p = 0.771). For both births and abortions, the proportion of pregnancies involving at least one prescription medication increased slightly from 2001 to 2013 (from 62.3% to 68.8% (p < 0.0001) and 42.6% to 46.8% (p<0.0001), respectively; **Fig 2A and Fig 2B**).

**Fig 3:**
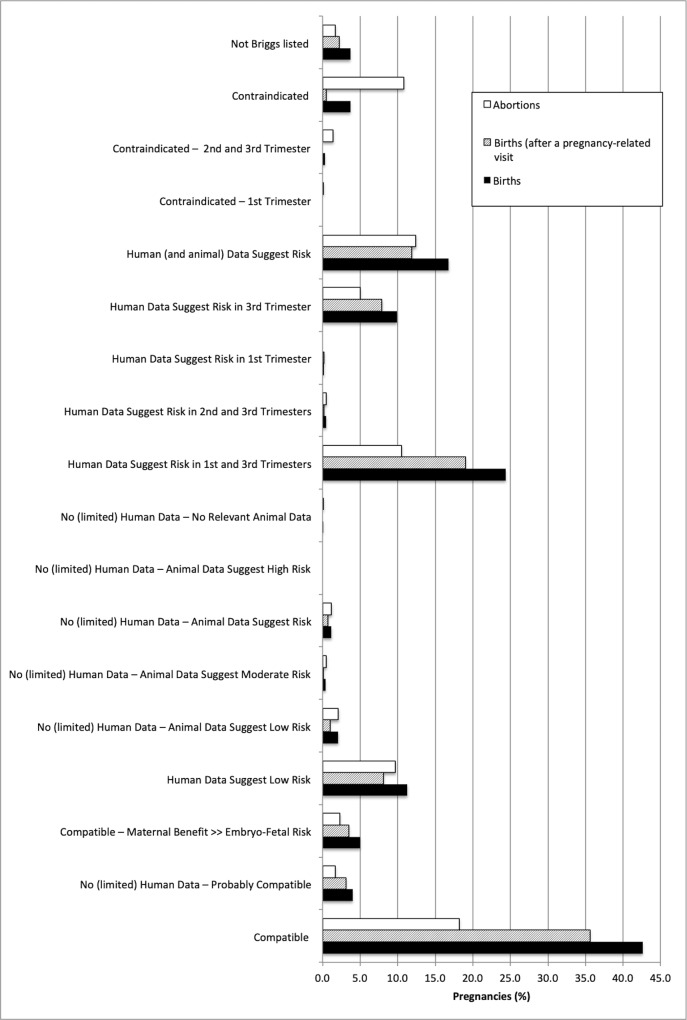
Pregnancies involving the receipt of at least one prescription medication, by pregnancy outcome and risk during pregnancy (2001–2013).

**Fig 4:**
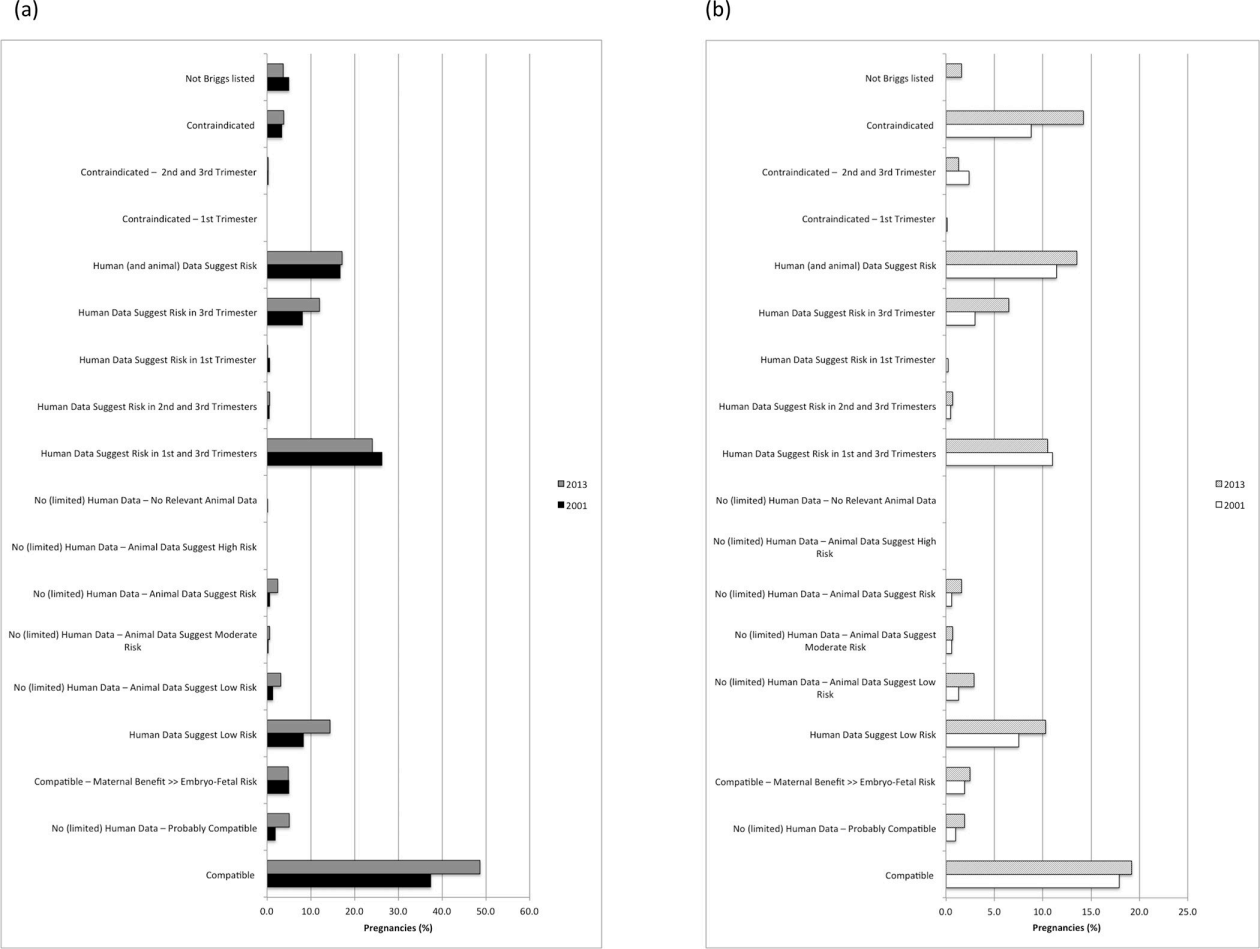
(a) Births (livebirth/stillbirth) and (b) Abortions involving the receipt of at least one prescription medication, by year and risk during pregnancy.

When medication exposure during pregnancy was examined by drug risk category, the most commonly used medications during pregnancy included those in the “Compatible” category (42.6% and 18.2% of births and abortions, respectively), followed by “Human data suggest risk in 1^st^ and 3^rd^ trimester” (24.4% and 10.5% of births and abortions, respectively) (**Fig 3**). In pregnancies that resulted in a live birth, more women were exposed to a medication before the first pregnancy-related visit than in the remaining pregnancy period after this first visit (p<0.0001). A greater proportion of pregnancies that ended in abortions were exposed to medications that are categorized as contraindicated (3.7% and 10.8% of births and abortions, respectively, p<0.0001). **Fig 4A** and **Fig 4B** compare the proportion of pregnancies involving at least one medication by risk category in 2001 and 2013 for births and abortions, respectively. An increase in the use of medications considered “Compatible” (Briggs category 1 in **S2 Table**), “Probably Compatible” (Briggs category 2, “No (limited) Human Data–Probably Compatible”) or low risk during pregnancy (Briggs category 4, “Human Data Suggest Low Risk”; and Briggs category 5, “No (limited) Human Data–Animal Data Suggest Low Risk”) was observed among births from 2001 to 2013 from 37.4% to 48.7% (p<0.0001) and 8.3% to 14.4% (p<0.0001), respectively (**Fig 4A**). However, a slight decrease in the use of medications categorized as “Human Data Suggest Risk in 1^st^ and 3^rd^ Trimesters” was observed for this population from 26.2% in 2001 to 24.0% in 2013 (p = 0.001). For pregnancies that resulted in abortion, the use of medications in this category also slightly decreased from 11.0% to 10.5% (p = 0.263) over the same time period (**Fig 4B**). Interestingly, the use of “Contraindicated” medications increased from 8.7% in 2001 to 15% in 2013 (p<0.0001) among the pregnancies that resulted in abortion, and increased only from 3.4% to 3.8% (p = 0.515) during the same time period among pregnancies that did not result in abortion.

The most common drugs used in pregnancy were amoxicillin, doxylamine, codeine combinations, nitrofurantoin, cephalexin, salbutamol and ranitidine (**Table 2**). A greater proportion of pregnancies that ended in abortion received a prescription for codeine, metronidazole, lorazepam, naproxen and citalopram than birth pregnancies.

**Table 2:** Most common drugs and their risk category filled among pregnant women who received at least one prescription.

Drug	Briggs Category	Pregnancies resulting in birth (n = 174,848)				Pregnancies resulting in abortion (N = 71,969)			
		Pregnancies with ≥ 1 prescription (N)	% (95% CI)	Pregnancies with ≥ 1 Rx after pregnancy-related visit (N)	% (95% CI)	Pregnancies with ≥1 prescription (N)	% (95% CI)	Pregnancies with ≥ 1 Rx after pregnancy-related visit (N)	% (95% CI)
Amoxicillin	Human Data Suggest Risk in 1st and 3rd Trimesters	37,811	21.62 (21.41–21.84)	30,842	17.64 (17.44–17.84)	4,159	5.78 (5.6–5.95)	961	1.34 (1.25–1.42)
Doxylamine	Compatible	33,804	19.33 (19.13–19.54)	26,799	15.33 (15.14–15.51)	4,210	5.85 (5.67–6.03)	1,651	2.29 (2.18–2.4)
Codeine, combinations	Human and animal data suggest risk	12,461	7.13 (7–7.25)	8,018	4.59 (4.49–4.69)	5,429	7.54 (7.34–7.74)	1,577	2.19 (2.08–2.3)
Nitrofurantoin	Human Data Suggest Risk in 3rd Trimester	12,372	7.08 (6.95–7.2)	10,374	5.93 (5.82–6.05)	1,158	1.61 (1.52–1.7)	362	0.5 (0.45–0.55)
Cefalexin	Compatible	11,353	6.49 (6.37–6.61)	9,259	5.3 (5.19–5.4)	1,388	1.93 (1.83–2.03)	470	0.65 (0.59–0.71)
Salbutamol	Compatible	8,185	4.68 (4.58–4.78)	6,581	3.76 (3.67–3.85)	1,620	2.25 (2.14–2.36)	240	0.33 (0.29–0.38)
Ranitidine	Compatible	6,415	3.67 (3.58–3.76)	5,559	3.18 (3.1–3.26)	562	0.78 (0.72–0.85)	94	0.13 (0.1–0.16)
Metronidazole	Human Data Suggest Low Risk	5,472	3.13 (3.05–3.21)	4,470	2.56 (2.48–2.63)	3,376	4.69 (4.53–4.85)	685	0.95 (0.88–1.02)
Azithromycin	Compatible	5,370	3.07 (2.99–3.15)	3,739	2.14 (2.07–2.21)	1,192	1.66 (1.56–1.75)	187	0.26 (0.22–0.3)
Hydrocortisone topical	Human (and animal) Data Suggest Risk	4,972	2.84 (2.76–2.92)	4,048	2.32 (2.24–2.39)	640	0.61 (0.55–0.67)	81	0.11 (0.09–0.14)
Betamethasone topical	Compatible—Maternal Benefit >> Embryo-Fetal Risk	4,794	2.74 (2.66–2.82)	3,472	1.99 (1.92–2.05)	437	0.89 (0.82–0.96)	92	0.13 (0.1–0.15)
Levothyroxine	Compatible	4,691	2.68 (2.61–2.76)	4,536	2.59 (2.52–2.67)	946	1.31 (1.23–1.4)	220	0.31 (0.27–0.35)
Erythromycin	Compatible	4,498	2.57 (2.5–2.65)	3,238	1.85 (1.79–1.92)	553	0.77 (0.7–0.83)	94	0.13 (0.1–0.16)
TMP/SMX	Human (and animal) Data Suggest Risk	3,898	2.23 (2.16–2.3)	2,194	1.25 (1.2–1.31)	951	1.32 (1.24–1.41)	117	0.16 (0.13–0.19)
Phenoxymethylpenicillin	Compatible	3,732	2.13 (2.07–2.2)	2,232	1.28 (1.22–1.33)	636	0.88 (0.82–0.95)	83	0.12 (0.09–0.14)
Labetalol	Human Data Suggest Low Risk	3,248	1.86 (1.79–1.92)	3,213	1.84 (1.77–1.9)	143	0.2 (0.17–0.23)	74	0.1 (0.08–0.13)
Acyclovir topical	Compatible	3,005	1.72 (1.66–1.78)	2,581	1.48 (1.42–1.53)	234	0.33 (0.28–0.37)	44	0.06 (0.04–0.08)
Hydrocortisone rectal	Human (and animal) Data Suggest Risk	2,966	1.7 (1.64–1.76)	2,737	1.57 (1.51–1.62)	133	0.18 (0.15–0.22)	39	0.05 (0.04–0.07)
Progesterone	Not Briggs listed	2,877	1.65 (1.59–1.71)	1,781	1.02 (0.97–1.07)	646	0.9 (0.83–0.97)	286	0.4 (0.35–0.44)
Clindamycin	Compatible	2,616	1.5 (1.44–1.55)	1,913	1.09 (1.05–1.14)	481	0.67 (0.61–0.73)	237	0.17 (0.14–0.2)
Fluticasone inhaled	Compatible	2,505	1.43 (1.38–1.49)	1,988	1.14 (1.09–1.19)	430	0.6 (0.54–0.65)	70	0.1 (0.07–0.12)
Lorazepam	Human Data Suggest Risk in 1st and 3rd Trimesters	2,440	1.4 (1.34–1.45)	1,409	0.81 (0.76–0.85)	1,124	1.56 (1.47–1.65)	237	0.33 (0.29–0.37)
Insulin (human)	Compatible	2,337	1.34 (1.28–1.39)	2,275	1.3 (1.25–1.35)	270	0.38 (0.33–0.42)	158	0.22 (0.19–0.25)
Mometasone inhaled	No (limited) Human Data—Probably Compatible	2,281	1.3 (1.25–1.36)	1,709	0.98 (0.93–1.02)	288	0.4 (0.35–0.45)	33	0.05 (0.03–0.06)
Naproxen	Human Data Suggest Risk in 1st and 3rd Trimesters	2,132	1.22 (1.17–1.27)	548	0.31 (0.3–0.3)	1,745	2.42 (2.31–2.54)	616	0.86 (0.79–0.92)
Cloxacillin	Compatible	2,083	1.19 (1.14–1.24)	1,356	0.8 (0.7–0.8)	312	0.4 (0.4–0.5)	45	0.06 (0.04–0.08)
Citalopram	Human Data Suggest Risk in 3rd Trimester	2,011	1.15 (1.1–1.2)	1,183	0.7 (0.6–0.7)	1,015	1.4 (1.3–1.5)	146	0.2 (0.17–0.24)
Fusidic acid topical	Not Briggs listed	1,750	1 (0.95–1.05)	1,130	0.7 (0.6–0.7)	292	0.4 (0.4–0.5)	41	0.06 (0.04–0.07)

TMP/SMX = trimethoprim/sulfamethoxazole

**Fig 5:**
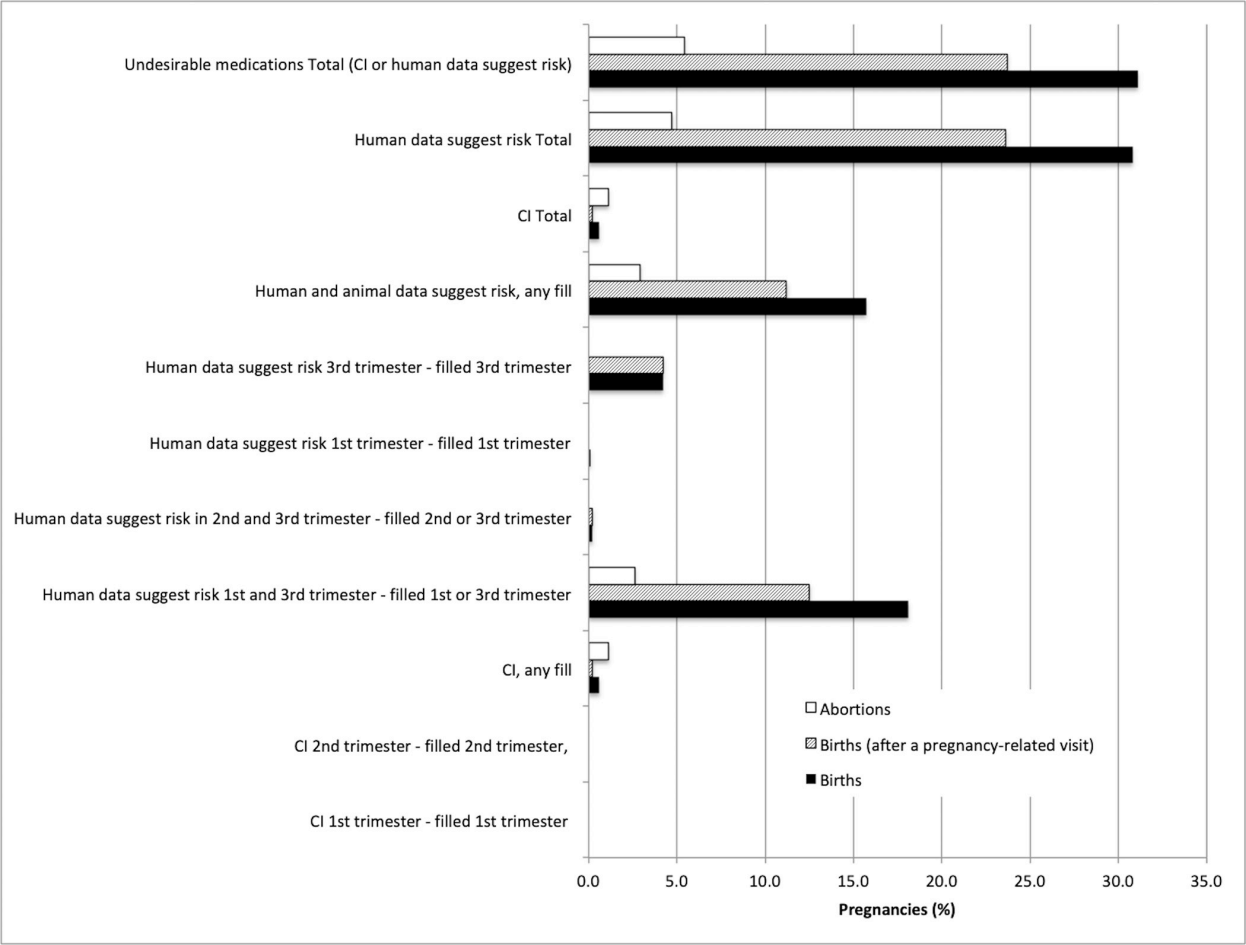
Pregnancies involving the receipt of at least one prescription for an undesirable medication during pregnancy, by risk category. *Excludes oral contraceptives, estrogens, progestogens, ovulation stimulants and leuprolide. CI–contraindicated.

**Fig 6:**
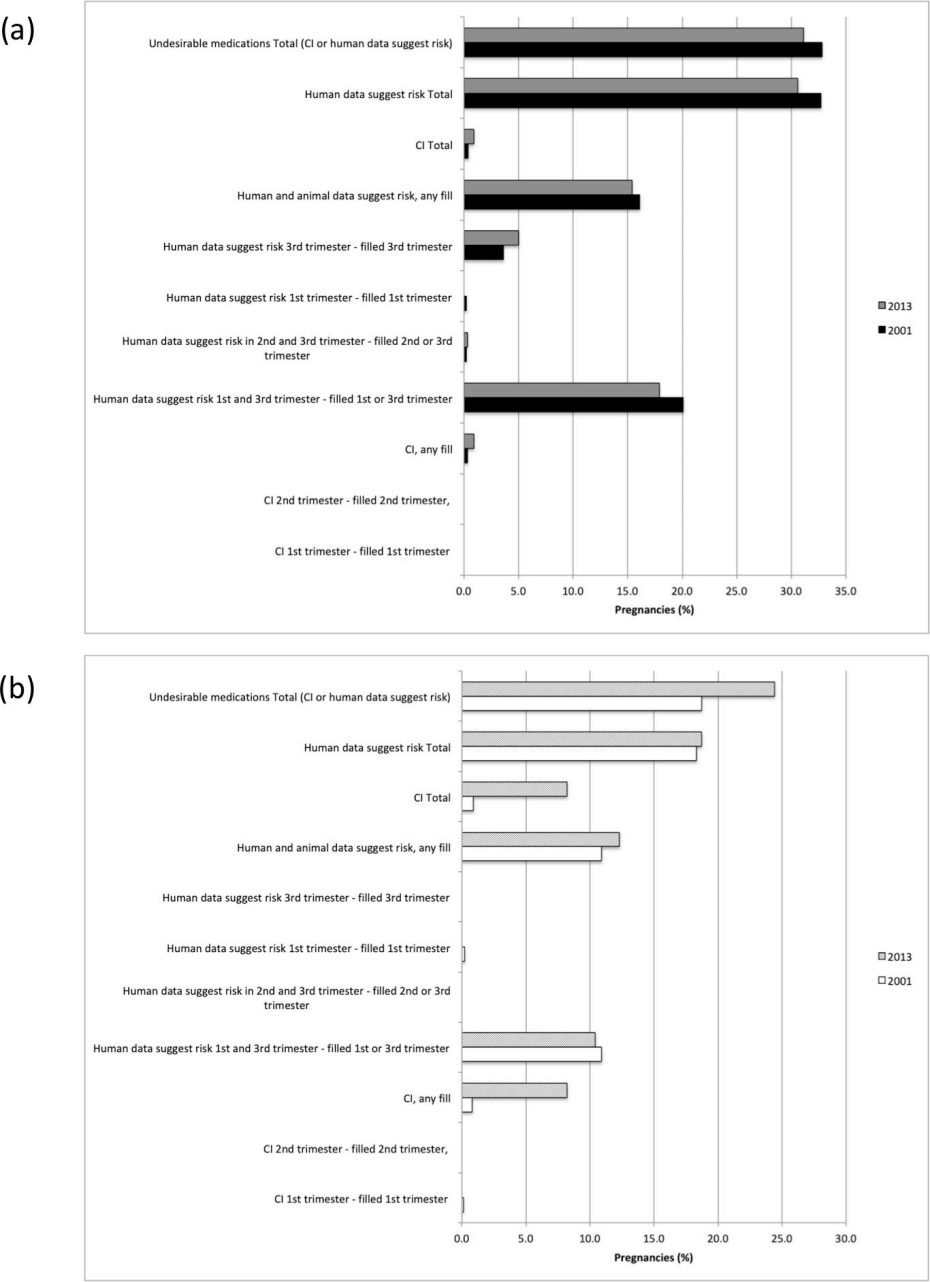
**(a) Births (livebirth/stillbirth) and (b) Abortions involving dispensation of at least one prescription for an undesirable medication, by year and risk during pregnancy.** Excludes oral contraceptives, estrogens, progestogens, ovulation stimulants and leuprolide; CI = contraindicated.

Again, a higher proportion of pregnancies that resulted in abortion had received a prescription for a contraindicated medication compared to pregnancies that resulted in birth regardless if the prescription was filled at any time or only after a pregnancy-related visit (p<0.0001) (**Fig 5**). However, a greater proportion of birth pregnancies filled a prescription for an undesirable medication after conception (31.1%), and this proportion dropped to 23.7% after a pregnancy-related visit (p<0.0001). Among birth pregnancies, there was a slight drop in the use of undesirable medications during pregnancy from 32.8% in 2001 to 31.1% in 2013 (p = 0.003) (**Fig 6A**). In contrast, there was an increase in the receipt of an undesirable medication among pregnancies that ended in abortion from 18.7% in 2001 to 24.4% in 2013 (p<0.0001) (**Fig 6B**). The most common medications used that are considered undesirable in pregnancy according to Briggs pregnancy recommendation categories 10 to 17^11^ (excluding female reproductive hormones) included amoxicillin and codeine combinations (**S3 Table**).

## Discussion

We found an increase in the proportion of pregnancies involving at least one medication from 2001 to 2013, with a higher proportion of pregnancies resulting in abortion exposed to a medication considered “Contraindicated” compared to pregnancies resulting in birth. We also observed a drop in the receipt of undesirable medication among birth pregnancies after a pregnancy-related health visit, which may indicate a decrease in the intentional use of medication in pregnancy.

The increase in the use of prescription medications over time that was observed in this study is similar to previously reported trends in pregnant women [5,19–23]. This could reflect a shift in the perceived risk of exposure for certain drugs to the fetus relative to the importance of maternal treatment and the availability of newer drugs into the market over time. While a higher proportion of pregnancies resulting in abortion were exposed to a contraindicated medication than birth pregnancies, it is uncertain as to whether the abortion was a result of the medication exposure or if the medication use was based on the knowledge that the pregnancy would be aborted. Furthermore, it is not possible to determine based on this data if the aborted pregnancy was intentional or spontaneous. If the abortion was spontaneous following medication exposure, this might be a reflection of the safety of the medication during pregnancy. However, if the abortion was intentional, there could have been less attention to the safety of medication use during an unwanted pregnancy. Interestingly, there appears to be a drop in medications being filled among pregnant women after a pregnancy-related visit. This could be due to the perceived risk of harm to the fetus or treatment no longer being needed.

We found nearly 65% of women with a pregnancy that resulted in birth took at least one prescription medication in pregnancy and 55% of women received a prescription after a pregnancy-related visit. Furthermore, 3.7% of births and 10.8% of abortions were exposed to a medication categorized as “Contraindicated” according to Briggs. The rate of drug use in pregnancy ranged from 27% to 93% for exposure to at least one prescription (excluding vitamins and minerals) and 0.9% to 4.5% for contraindicated medications in previous studies [3]. In a recent prospective study of 9,546 nulliparous women by Haas, et al., 73% of women took at least one medication during pregnancy and 55% took a medication in the first trimester. Similar to previous large population-based [1–9] and survey-based studies [24–28], antiinfectives for systemic use was among the most common class of medications used during pregnancy in the present study. Haas et al. found gastrointestinal agents, antibiotics, and analgesics were the most common medication classes taken in pregnancy. The slight differences in rates of medication use between our study and the study by Haas, et al. is likely attributed to the inclusion of over-the-counter medications and the prospective interview-based design to capture all medication use in the study by Haas, et al. [28] However, both the present study and the study by Haas et al. highlight the potential exposure to medications during an important period in fetal development in the first trimester, particularly when the receipt of medication was unintentional or prior to knowledge of pregnancy.

Not surprisingly, amoxicillin and doxylamine were the two most common medications filled during pregnancy, which was also reported by Smolina et al. [5]. Amoxicillin and codeine were the two most common undesirable medications filled during pregnancy. Of note, the risk category for amoxicillin changed from “Compatible” to “Risk in first and third trimester”, which contributed to the increase in the number of women included in the “drugs not recommended in pregnancy” group. This change was based on a 2012 observational study that observed a low but increased risk of cleft lip with/without cleft palate for infants exposed to amoxicillin in the first trimester (adjusted odds ratio (OR) 2.0 (95% CI 1.0–4.1)) and third gestational month (OR 4.3 (95% CI 1.4–13)) between 1994 and 2008 [29]. The risk of cleft palate alone was OR 1.0 (95% CI 0.4–2.3) and OR 7.1 (95% CI 1.4–36) for the two exposure periods, respectively. Of note, the observation period in the current study (2001–2013) is reflective of a time when amoxicillin was not considered to be of risk.

The strengths of our study include the ability to study a large population of all residents in Manitoba with a comprehensive database not restricted to income, age, or drug insurance coverage. We also were able to identify the rates of drug use after a pregnancy-related visit to capture those who filled the prescription knowing that they were pregnant. We also reported on pregnancies that resulted in abortion in addition to pregnancies that resulted in livebirth or stillbirth, which is a limitation in previous studies^5^. Moreover, we examined medication use by Briggs category, which may provide more clinical insight into risk by pregnancy timing than the FDA categories. The successive FDA categories do not necessarily mean increasing severity of risk.

There were limitations of our study that warrant discussion. The use of administrative data for this study only captured those who used medical services during pregnancy, and only included hospital births. However, only 0.8% of deliveries in Manitoba have been reported to occur at home with a midwife [16]. The majority of abortions in Manitoba occur in hospitals (61.2%) [17], and mifepristone was not yet available in Canada at the time of the study, although it is possible that some abortions captured in physician billing claims only were not captured. However, this study is unique in capturing medication use during abortions in a Canadian population. Like most pharmacoepidemiology studies using administrative data, drug dispensions may not always reflect drug consumption and we only reported on drugs filled in an outpatient community pharmacy. Furthermore, the DPIN does not consistently capture over-the-counter medications and therefore was excluded in the current analysis, even if there were prescriptions for over the counter medications used in pregnancy. Although many women take over the counter medications in pregnancy, without an ability to quantify use though prescription filling, we felt that it was more accurate to exclude these medications. Pregnancy-related visits were used as an indicator that the patient had knowledge of the pregnancy. However, because home pregnancy tests are not captured through administrative claims data, these visits may not reflect the very first knowledge of pregnancy or the start of pregnancy. It is also important to note that the average time at risk of a medication use after a pregnancy-related visit was shorter for abortions. Of note, since conception usually occurs approximately two weeks after the last menstrual period, our estimation of conception may actually be an approximation of the last menstrual period rather than true conception of pregnancy. Medication taking behavior prior to an abortion, perception of risk, and many other factors are likely important to consider in a holistic approach to medication use during aborted pregnancies, this information is not captured in administrative health claims data. We did not conduct analyses based on spontaneous or induced abortions, although medication-taking behavior in these two situations are likely different. There were more estimated gestational ages for abortions and potential for misclassification, however, we attempted a conservative window of exposure to medications during these pregnancies to avoid misclassification of a mother taking a medication but not being pregnant yet. We were also not able to capture medications received in hospital, however, antenatal hospitalization in Manitoba (without delivery) in 2008/09 was 11 per 100 [16], and any discharge prescriptions would be captured through DPIN. Our findings may only be generalizable to jurisdictions with a similar drug coverage program in which eligible prescriptions are covered for Manitoba residents by the province after an income-based deductible is reached per year. Finally, future work could investigate whether certain medication classes or medications contributed to the overall trend in medication use during pregnancy.

## Conclusion

There was an increase in the proportion of pregnancies involving at least one medication from 2001 to 2013. Few women fill prescriptions for medications undesirable during pregnancy after a pregnancy-related visit. Contraindicated medications in pregnancy were dispensed to a higher proportion of pregnancies resulting in abortion compared to pregnancies resulting in a birth. We observed a drop in the receipt of medication among birth pregnancies after a pregnancy-related health visit. When describing intentional use of medication during pregnancy, it is important to consider prescriptions filled after the first pregnancy-related visit.

## Supporting information

S1 Table Pregnancies during which at least 1 prescription filled, by World Health Organization Anatomical Therapeutic Chemical Level 1 (ATC1) classification and study period. Weighted total pregnancies = 191,222.06 (N, weighted %, 95% CI)
(DOCX)

S2 Table Briggs categories. *Medications under this category were considered undesirable during pregnancy
(DOCX)

S3 Table Pregnancies involving the receipt of at least one prescription for undesirable medication during pregnancy. Excludes oral contraceptives, estrogens, progestogens, ovulation stimulants and leuprolide, *Prescription filled in first trimester **Prescription filled in third trimester, S = Suppressed (value is <6)
(DOCX)
