# Supplementary material for: Prescription medication use during pregnancies that resulted in births and abortions (2001-2013): A retrospective population-based study in a Canadian population
Source: PLoS One. 2019 Mar 6;14(3):e0211319. doi: 10.1371/journal.pone.0211319 (PMC6402756; doi:10.1371/journal.pone.0211319)
Supplement: S1 Table — Weighted total pregnancies = 191,222.06 (N, weighted %, 95% CI) (DOCX) [file pone.0211319.s001.docx]

**S1 Table**. Pregnancies during which at least 1 prescription filled, by World Health Organization Anatomical Therapeutic Chemical Level 1 (ATC1) classification and study period. Weighted total pregnancies = 191,222.06

|  |  | Pregnancy Overall  (N, weighted %, 95% CI) | Pregnancy (Rx after pregnancy-related visit)  (N, weighted %, 95% CI) |
| --- | --- | --- | --- |
| ATC1 | ATC1 Name |  |  |
| A | ALIMENTARY TRACT AND METABOLISM | 18614 (9.73)  (95% CI 9.59-9.87) | 13488 (7.05)  (95% CI 6.93-7.17) |
| B | BLOOD AND BLOOD FORMING ORGANS | 6324 (3.31)  (95% CI 3.23-3.39) | 4618 (2.41)  (95% CI 2.35-2.48) |
| C | CARDIOVASCULAR SYSTEM | 10096 (5.28)  (95% CI 5.18-5.38) | 8476 (4.43)  (95% CI 4.34-4.53) |
| D | DERMATOLOGICALS | 19962 (10.44)  (95% CI 10.29-10.58) | 13877 (7.26)  (95% CI 7.14-7.38) |
| G | GENITO URINARY SYSTEM AND SEX HORMONES | 19110 (9.99)  (95% CI 9.85-10.14) | 6277 (3.28)  (95% CI 3.20-3.36) |
| H | SYSTEMIC HORMONAL PREPARATIONS, | 8570 (4.48)  (95% CI 4.39-4.58) | 6271 (3.28)  (95% CI 3.20-3.36) |
| J | ANTIINFECTIVES FOR SYSTEMIC USE | 79307 (41.47)  (95% CI 41.19-41.76) | 56688 (29.65)  (95% CI 29.40-29.89) |
| L | ANTINEOPLASTIC AND IMMUNOMODULATING AGENTS | 785 (0.41)  (95% CI 0.38-0.44) | 178 (0.09)  (95% CI 0.08-0.11) |
| M | MUSCULO-SKELETAL SYSTEM | 7039 (3.68)  (95% CI 3.60-3.77) | 2157 (1.13)  (95% CI 1.08-1.18) |
| N | NERVOUS SYSTEM | 32866 (17.19)  (95% CI 17.00-17.37) | 17193 (8.99)  (95% CI 8.86-9.13) |
| P | ANTIPARASITIC PRODUCTS, INSECTICIDES AND REPELLENTS | 9181 (4.80)  (95% CI 4.70-4.90) | 5322 (2.78)  (95% CI 2.71-2.86) |
| R | RESPIRATORY SYSTEM | 50137 (26.22)  (95% CI 25.99-26.45) | 36981 (19.34)  (95% CI 19.14-19.54) |
| S | SENSORY ORGANS | 5531 (2.89)  (95% CI 2.82-2.97) | 3355 (1.75)  (95% CI 1.70-1.81) |
| V | VARIOUS | 12 (0.01)  (95% CI 0.00-0.01) | s* |

*s represents <6 people to comply with privacy policy
