# Supplementary material for: Prescription medication use during pregnancies that resulted in births and abortions (2001-2013): A retrospective population-based study in a Canadian population
Source: PLoS One. 2019 Mar 6;14(3):e0211319. doi: 10.1371/journal.pone.0211319 (PMC6402756; doi:10.1371/journal.pone.0211319)
Supplement: S2 Table — *Medications under this category were considered undesirable during pregnancy (DOCX) [file pone.0211319.s002.docx]

**S2 Table**. Briggs Categories

| 1 | Compatible |
| --- | --- |
| 2 | No (limited) Human Data – Probably Compatible |
| 3 | Compatible – Maternal Benefit >> Embryo-Fetal Risk |
| 4 | Human Data Suggest Low Risk |
| 5 | No (limited) Human Data – Animal Data Suggest Low Risk |
| 6 | No (limited) Human Data – Animal Data Suggest Moderate Risk |
| 7 | No (limited) Human Data – Animal Data Suggest Risk |
| 8 | No (limited) Human Data – Animal Data Suggest High Risk |
| 9 | No (limited) Human Data – No Relevant Animal Data |
| 10 | Human Data Suggest Risk in 1^st^ and 3^rd^ Trimesters* |
| 11 | Human Data Suggest Risk in 2^nd^ and 3^rd^ Trimesters* |
| 12 | Human Data Suggest Risk in 1^st^ Trimester* |
| 13 | Human Data Suggest Risk in 3^rd^ Trimester* |
| 14 | Human (and animal) Data Suggest Risk* |
| 15 | Contraindicated – 1^st^ Trimester* |
| 16 | Contraindicated – 2^nd^ and 3^rd^ Trimester* |
| 17 | Contraindicated* |
| 99 | Not Briggs listed |

*Medications under this category were considered undesirable during pregnancy
