# Supplementary material for: Prescription medication use during pregnancies that resulted in births and abortions (2001-2013): A retrospective population-based study in a Canadian population
Source: PLoS One. 2019 Mar 6;14(3):e0211319. doi: 10.1371/journal.pone.0211319 (PMC6402756; doi:10.1371/journal.pone.0211319)
Supplement: S3 Table — Excludes oral contraceptives, estrogens, progestogens, ovulation stimulants and leuprolide, *Prescription filled in first trimester **Prescription filled in third trimester, S = Suppressed (value is <6) (DOCX) [file pone.0211319.s003.docx]

**S3 Table.** Most common undesirable medication filled during pregnancy among pregnant women who received at least one prescription during pregnancy (N,%)

| Drug | Briggs Category | Pregnancies resulting in births (n=174,848) | Pregnancies resulting in abortion  (N=71,969) |
| --- | --- | --- | --- |
| Amoxicillin | Human Data Suggest Risk in 1st and 3rd Trimesters | 14,078 (8.1)*  15,613 (8.9)** | 4,159 (5.8)*  0 (0)** |
| Codeine, combinations | Human (and animal) Data Suggest Risk | 12,461 (7.1) | 5,429 (7.5) |
| Nitrofurantoin | Human Data Suggest Risk in 3rd Trimester | 4631 (2.7)** | 0 (0)** |
| Hydrocortisone topical | Human (and animal) Data Suggest Risk | 4,972 (2.8) | 438 (0.6) |
| TMP/SMX | Human (and animal) Data Suggest Risk | 3,898 (2.2) | 651 (1.3) |
| Hydrocortisone rectal | Human (and animal) Data Suggest Risk | 2,966 (1.7) | 16 (0) |
| Lorazepam | Human Data Suggest Risk in 1st and 3rd Trimesters | 1458 (0.8)*  827 (0.5) ** | 1105 (1.5)*  0 (0)** |
| Naproxen | Human Data Suggest Risk in 1st and 3rd Trimesters | 1650 (0.9)*  287 (0.2)** | 1711 (2.4)*  0 (0)** |
| Citalopram | Human Data Suggest Risk in 3rd Trimester | 923 (0.5)** | 0 (0)** |
| Venlafaxine | Human Data Suggest Risk in 3rd Trimester | 723 (0.4)** | 0 (0)** |
| Amoxicillin and enzyme inhibitor | Human Data Suggest Risk in 1st and 3rd Trimesters | 634 (0.4)*  474 (0.3)** | 309 (0.4)*  0 (0)** |
| Prednisone | Human (and animal) Data Suggest Risk | 1439 (0.8) | 325 (0.5) |
| Paroxetine | Human (and animal) Data Suggest Risk | 1325 (0.8) | 617 (0.9) |
| Sertraline | Human Data Suggest Risk in 3rd Trimester | 634 (0.4)** | 0 (0)** |
| Alprazolam | Human (and animal) Data Suggest Risk | 1007 (0.6) | 550 (0.8) |
| Fluoxetine | Human Data Suggest Risk in 3rd Trimester | 457 (0.3)** | 0 (0)** |
| Ibuprofen | Human Data Suggest Risk in 1st and 3rd Trimesters | 342 (0.2)*  185 (0.1)** | 169 (0.2)*  0 (0)** |
| Hydrocortisone acetate (rectal) | Human (and animal) Data Suggest Risk | 567 (0.3) | 88 (0.2) |
| Diclofenac | Human Data Suggest Risk in 1st and 3rd Trimesters | 421 (0.2)*  52 (0)** | S*  0 (0)** |
| Epinephrine (injectable) | Human (and animal) Data Suggest Risk | 519 (0.3) | 67 (0.1) |
| Diazepam | Human Data Suggest Risk in 1st and 3rd Trimesters | 322 (0.2)*  151 (0.1)** | 214 (0.3)*  0 (0)** |
| Triamcinolone and antibiotics (topical) | Human (and animal) Data Suggest Risk | 421 (0.2) | 46 (0.1) |
| Ampicillin | Human (and animal) Data Suggest Risk in 1^st^ Trimester | 105 (0.1)* | 24 (0.1)* |
| Paracetamol, combinations excl. psycholeptics | Human (and animal) Data Suggest Risk | 365 (0.2) | 238 (0.3) |

Excludes oral contraceptives, estrogens, progestogens, ovulation stimulants and leuprolide

*Prescription filled in first trimester **Prescription filled in third trimester, S=Suppressed (value is <6)
